# Impact of extent of coronary artery disease and percutaneous revascularization assessed by the SYNTAX score on outcomes following transcatheter aortic valve replacement

**DOI:** 10.1186/s12872-021-02374-y

**Published:** 2021-11-30

**Authors:** Tilman Stephan, Eva Thoma, Manuel Rattka, Dominik Felbel, Dominik Buckert, Wolfgang Rottbauer, Birgid Gonska, Sinisa Markovic

**Affiliations:** Department of Cardiology, Angiology, Pneumology and Internal Intensive Care, University Hospital Ulm, University of Ulm, Albert-Einstein-Allee 23, 89081 Ulm, Germany

**Keywords:** Aortic valve disease, Aortic stenosis, Coronary artery disease, Transcatheter aortic valve replacement, Percutaneous valve therapy, Percutaneous coronary intervention

## Abstract

**Objectives:**

The aim of the study was to analyze the impact of concomitant coronary artery disease (CAD) assessed by the SYNTAX score (SS) and periprocedural percutaneous coronary intervention (PCI) on outcomes after transcatheter aortic valve replacement (TAVR).

**Background:**

Due to controversial data regarding the effect of CAD on outcomes after TAVR, proper revascularization strategies remain a matter of debate.

**Methods:**

553 patients with severe aortic stenosis undergoing TAVR were included in this study. SS was calculated for each patient at baseline and after PCI. Primary outcome was one-year all-cause mortality.

**Results:**

60.2% of patients (N = 333) exhibited CAD with a mean SS of 10.8 ± 8.8. Of those, 120 patients (36.0%) received periprocedural PCI. In the treatment group, mean SS was decreased from 14.9 ± 9.1 to 6.3 ± 6.7. Patients with concomitant CAD suffered more frequently from myocardial infarction (MI) post TAVR compared to those without CAD (2.1% vs. 0.0%; *P* < 0.01). In the CAD cohort, MI rates were comparable between patients with and without PCI (2.2% vs. 2.5%; *P* = 0.71). Regarding SS, patients with a residual SS < 8 showed significant lower rates of one-year mortality (9.0% vs. 18.2%; *P* = 0.016) and MACCE (16.5% vs. 32.2%; *P* = 0.001). Besides left bundle brunch, predictors for an increased one-year mortality were a residual SS ≥ 8 in the CAD group (OR = 3.17; *P* = 0.011) and a EuroSCORE ≥ 4% in the entire study population (OR = 2.18; *P* = 0.017).

**Conclusion:**

Our results suggest that a residual SS-guided revascularization strategy may improve prognosis after TAVR in patients with concomitant CAD. PCI aiming for a residual SS < 8 was associated with improved one-year clinical outcomes.

**Supplementary Information:**

The online version contains supplementary material available at 10.1186/s12872-021-02374-y.

## Introduction

Concomitant coronary artery disease (CAD) is highly prevalent among patients with severe aortic stenosis (AS) [1–3]. The common clinical occurrence is related at least in part to similarities in risk factors and pathogenesis [4, 5]. Until recently, surgical aortic valve replacement (SAVR) with concomitant coronary artery bypass grafting (CABG) has been the main treatment strategy for patients with severe AS and significant CAD [6]. In this context, it is well known that additional CAD increases the risk for perioperative complications and impairs long-term outcomes after SAVR [7–9]. However, combined SAVR and CABG lead to improved short- and long-term survival in patients with severe AS and CAD compared to those undergoing isolated SAVR [10–12]. Nowadays, transcatheter aortic valve replacement (TAVR) developed to an at least equivalent or even superior treatment option for severe AS, especially in higher risk and inoperable patients (class I level B recommendation) and has risen steadily year by year [13]. However, controversial results have been reported regarding the effect of CAD as well as of the impact of periprocedural percutaneous revascularization on clinical outcomes post TAVR [14]. This may be explained amongst others by the heterogeneous nature of CAD and the extent of revascularization. Therefore, some recent studies have classified TAVR patients according to CAD severity, mainly using the SYNTAX (Synergy Between PCI With Taxus and Cardiac Surgery) score (SS) [15]. Due to expanding indications for TAVR towards intermediate-risk and younger patients with consequently longer life expectancy, the assessment of the impact of CAD and its management has become more and more important.

The purpose of the present study was to analyze the impact of CAD, its severity and periprocedural percutaneous coronary intervention (PCI) assessed by the SYNTAX score on clinical outcomes after TAVR.

## Methods

This retrospective single-center study included 553 patients with symptomatic severe aortic valve stenosis undergoing TAVR at our university hospital center between 01/2010 and 12/2015. Severe AS was documented by echocardiography and cardiac catheterization with an aortic valve area (AVA) ≤ 1.0 cm^2^ or an indexed AVA ≤ 0.6 cm^2^/m^2^. Percutaneous cardiac catheterization was performed in all patients prior to TAVR. CAD was diagnosed in case of visually estimated coronary lesions with ≥ 50% lumen obstruction in at least one major epicardial coronary artery, previous revascularization procedure (either by PCI or CABG) or documented myocardial infarction. Obstructive CAD was defined as the presence of at least one lesion > 70% in one major coronary vessel (or > 50% for left main). Patients were referred for TAVR according to the interdisciplinary decision by the local institutional Heart Team consisting of invasive cardiologists and cardiovascular surgeons. The indication for TAVR was based on patients´ clinical history, clinical status, anatomical suitability, geriatric assessment, appropriate risk scores (STS score (Society of Thoracic Surgeons), EuroSCORE II (European System for Cardiac Operative Risk Evaluation) and the volition of the patient. In case of existing CAD, the interdisciplinary Heart Team also adjudicated on percutaneous coronary revascularization or conservative strategy in accordance with current revascularization guidelines and in consistence with the patient´s decision. Baseline SS was one of the most important criteria for decision-making. TAVR procedures were performed via the femoral access route using the Medtronic CoreValve bioprothesis (Medtronic, Minneapolis, MN, USA) or the Edwards Sapien 3 transcatheter heart valve (Edwards LiveSciences, Irvine, CA, USA). Clinical follow-up was routinely performed at 1, 3, 6 and 12 months after TAVR by means of a clinical visit in our cardiological outpatient clinic or during a follow-up hospitalization.

**Fig. 1:**
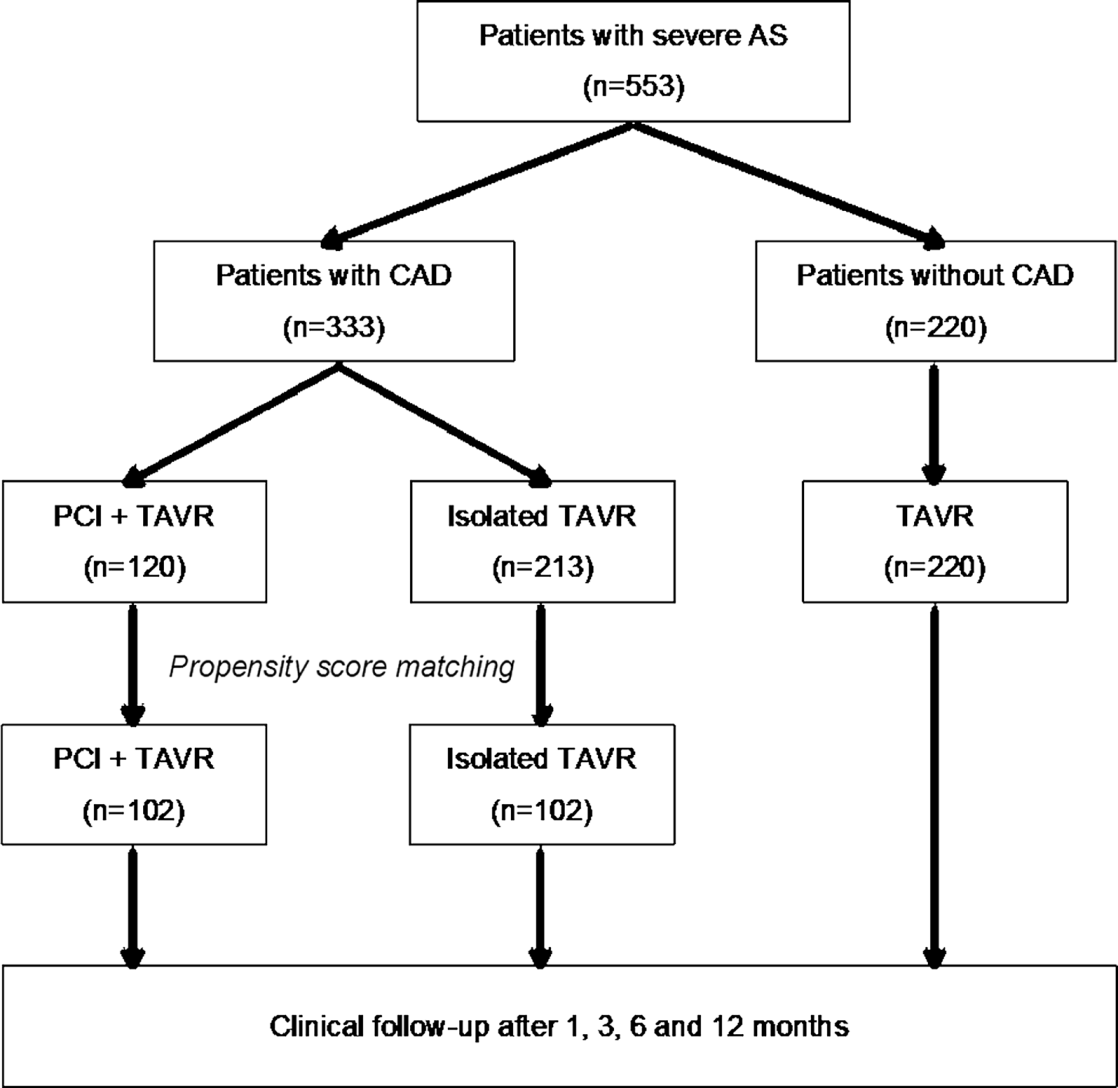
Study flow chart. *AS* aortic stenosis; *CAD* coronary artery disease; *PCI* percutaneous coronary intervention; *TAVR* transcatheter aortic valve replacement

Baseline clinical, echocardiographic, and procedural characteristics (for TAVR and PCI) were recorded for all enrolled patients and entered into an institutional database. Patients with a history of CABG were excluded from this analysis. The study population was dichotomized in patients with and without concomitant CAD. Patients with CAD were further divided into patients undergoing periprocedural PCI and patients treated conservatively (Fig. 1). PCI had to be performed within a period of 6 months prior and 3 months following TAVR. All procedures were performed according to contemporary guidelines, and all treatment decisions, including type of stent implanted or antithrombotic regimen were at the operator's discretion. SS was calculated for each patient at baseline—defined as SS prior to any PCI—using the SYNTAX score calculator [16] and used to assess the complexity of CAD. Additionally, in patients undergoing PCI as part of TAVR management, residual SS was calculated and defined as SS of remaining CAD after PCI. In patients not undergoing PCI, residual SS was considered equivalent to baseline SS.

The primary endpoint of this study was one-year all-cause mortality. Secondary endpoints were major adverse cardiac and cerebrovascular events (MACCE) within the first year following TAVR including death, myocardial infarction (MI), stroke, target lesion revascularization (TLR) and hospitalization due to decompensated heart failure as well as its individual components. Myocardial infarction was defined according to the current guidelines of myocardial infarction [17]. TLR was defined as any repetitive revascularization for restenosis at the lesion treated during index PCI.

The study was approved by the local ethics committee and has been performed in accordance with the ethical standards laid down in the Declaration of Helsinki. All patients provided written informed consent to participate in the ULM TAVR-Registry with subsequent follow-up assessment.

## Statistical analysis

Categorical data are presented as counts and percentages (%). Comparison of proportions was carried out using the χ^2^-test. Continuous variables are presented as mean ± one standard deviation (SD). Continuous variables for two groups were compared with the unpaired U-test.

Time-to-event analyses were performed using Kaplan–Meier (KM) estimates and were compared with the log-rank test. KM curves were generated for time to mortality.

To adjust for differences of baseline characteristics, a propensity score matching was performed for patients with and without periprocedural PCI. The variables chronic obstructive pulmonary disease (COPD), aortic valve area and age, showing statistically significant differences or a strong statistically trend between the two groups in the initial unmachted analysis, were employed in propensity score matching. A one-to-one matched analysis was performed. Patients were eligible for matching, if the difference of the estimated propensity score between PCI and no PCI was within the caliper radius of 0.10 * sigma.

Cox multivariate regression analysis was used to determine significant predictors of one-year mortality among patients with AS and CAD as well as for the entire study population. Models were developed with stepwise techniques and by consideration of variables that were clinically relevant. The following variables were included in the model: SS baseline, SS residual, SS difference, EuroSCORE II, aortic valve area, severe heart failure (left ventricular ejection fraction [LVEF] < 30%), severe pulmonary arterial hypertension, periprocedural PCI, left bundle branch block after TAVR (LBBB), Troponin T, serum creatinine, age and CAD. The strength of the association with mortality was estimated by calculating the adjusted hazard ratio (HR) with the 95% confidence interval.

A *P*-value of < 0.05 was considered to indicate statistical significance. Statistical analyses were calculated with Statistica release 7.1 software (StatSoft Inc., Tulsa, Oklahoma, USA). Propensity-score matching was performed with XLSTAT (XLSTAT-Premium, Addinsoft, New York, USA).

## Results

### Effect of CAD on outcomes after TAVR

#### Baseline characteristics

The present study included 553 patients with symptomatic severe AS undergoing TAVR via the femoral access (Table 1). Mean age was 81.1 ± 6.2 years and 43.8% of the patients were male. Mean baseline SS was 6.5 ± 8.7, STS score 6.9 ± 5.0 and EuroSCORE II 5.8 ± 4.9%. Mean aortic valve area using the Gorlin formula was 0.6 ± 0.2 cm^2^. In terms of functional status mean NYHA (New York Heart Association) class was 3.0 ± 0.7 and CCS (Canadian Cardiovascular Society) class 1.1 ± 1.4.

**Table 1:** Baseline characteristics of the entire study population as well as of patients with and without CAD separately

	Overall population (n = 553)	CAD (n = 333)	No CAD (n = 220)	*P* value
Age (years)	81.1 ± 6.2	81.4 ± 6.1	80.6 ± 6.4	0.41
Male (%)	242 (43.8)	160 (48.0)	82 (37.3)	**0.01**
Body mass index (BMI; kg/m^2^)	26.6 ± 4.7	26.6 ± 4.9	26.5 ± 4.5	0.73
NYHA functional class	3.0 ± 0.7	3.0 ± 0.8	3.1 ± 0.7	0.47
CCS class	1.1 ± 1.4	1.1 ± 1.4	1.0 ± 1.4	0.71
Diabetes mellitus (%)	160 (28.9)	98 (29.4)	62 (28.2)	0.75
Prior myocardial infarction (%)	73 (13.2)	73 (21.9)	0 (0.0)	** < 0.001**
Previous TIA/stroke (%)	69 (12.5)	42 (12.6)	27 (12.3)	0.91
Carotid stenosis > 70% (%)	24 (4.3)	17 (5.1)	7 (3.2)	0.27
Atrial fibrillation (%)	236 (42.7)	138 (41.4)	98 (44.6)	0.47
COPD (%)	301 (54.4)	180 (54.1)	121 (55.5)	0.77
Severe PAH (mmHg)	118 (21.3)	67 (20.1)	51 (23.5)	0.36
mPAP (mmHg)	30.4 ± 11.9	30.0 ± 11.4	31.1 ± 12.7	0.57
LVEF (%)	61.6 ± 21.5	61.7 ± 22.1	61.6 ± 20.7	0.61
Cardiac output (l/min)	4.5 ± 6.6	4.8 ± 8.3	4.1 ± 1.1	0.80
Severe heart failure (%)	35 (6.3)	21 (6.3)	14 (6.4)	0.97
PCWP (mmHg)	21.7 ± 10.3	22.0 ± 9.9	21.4 ± 10.9	0.64
Troponin T (ng/l)	72.7 ± 495	93.3 ± 646	43.9 ± 68	0.30
Renal insufficiency (%)	22 (40.1)	140 (42.0)	82 (37.3)	0.26
Creatinine level (mg/dl)	1.2 ± 0.7	1.2 ± 0.7	1.2 ± 0.7	0.24
Creatinine clearance (ml/min)	51.9 ± 19.7	51.8 ± 19.7	51.9 ± 19.7	0.95
Aortic valve area (cm^2^)	0.6 ± 0.2	0.6 ± 0.22	0.6 ± 0.2	0.71
MTPG (mmHg)	36.4 ± 15.7	35.1 ± 14.7	38.5 ± 17.2	0.11
EuroSCORE II (%)	5.8 ± 4.9	6.2 ± 5.3	5.2 ± 4.1	**0.02**
STS score	6.9 ± 5.0	6.9 ± 4.7	6.8 ± 5.3	0.51
SYNTAX score baseline	6.5 ± 8.7	10.8 ± 8.8	0.0	** < 0.001**

Data are mean ± standard deviation or counts (%).* P*-values set in boldface indicate statistical significance

*CCS* Canadian Cardiovascular Society; *COPD* chronic obstructive pulmonary disease; *EuroSCORE* European System for Cardiac Operative Risk Evaluation; *LVEF* left ventricular ejection fraction; *mPAP* mean pulmonary arterial pressure; *mTPG* mean transaortic pressure gradient; *NYHA* New York Heart Association; *PAH* pulmonary arterial hypertension; *PCWP* pulmonary capillary wedge pressure; *STS* Society of Thoracic Surgeons; *TIA* transient ischemic attack

CAD was diagnosed in 333 patients (60.2%). 120 patients (21.7% of the entire study population and 36.0% of patients with CAD, respectively) underwent periprocedural PCI as part of the TAVR management. Most baseline variables were comparable between patients with and without CAD and are presented in Table 1. The only significant difference was that patients with CAD were more frequently male (48.0% vs. 37.3%; *P* = 0.01). Cardiovascular risk factors and comorbidity burden were similar distributed between the two groups. Not surprisingly, mean EuroSCORE II was higher in patients with CAD compared to those without CAD (6.2 ± 5.3% vs. 5.2 ± 4.1%; *P* = 0.02). 73 patients in the CAD group (21.9%) had a history of MI. Mean baseline SS in the CAD cohort was 10.8 ± 8.8.

#### Clinical outcomes

One-year clinical outcomes of the entire cohort as well as of patients with and without CAD are displayed in Table 2. Overall, there were 76 deaths within the first year after TAVR (13.7%). No statistically significant risk difference was found for one-year all-cause mortality between patients with and without CAD (12.3% vs. 15.9%; *P* = 0.23). Similar findings were also observed across the two groups with respect to rates of MACCE (22.2% vs. 18.6; *P* = 0.31), stroke (1.8% vs. 1.4%; *P* = 0.69) and hospitalization due to decompensated heart failure (2.7% vs. 1.4%; *P* = 0.28) within the first year following TAVR. In patients with CAD, we observed significant higher rates of myocardial infarction compared to those without CAD (2.1% vs. 0.0%; *P* < 0.001). Furthermore, these patients required more often target lesion revascularization within the first year (3.3% vs. 0.0%; *P* < 0.001).

**Table 2:** One-year clinical outcomes after TAVR of the entire study population as well as of patients with and without CAD separately

	Overall population (n = 553)	CAD (n = 333)	No CAD (n = 220)	*P* value
SYNTAX score residual	4.3 ± 6.7	7.2 ± 7.3	0.0	** < 0.01**
One-year mortality	76 (13.7)	41 (12.3)	35 (15.9)	0.23
30-day mortality	16 (2.9)	11 (3.3)	5 (2.3)	0.47
MACCE	115 (20.8)	74 (22.2)	41 (18.6)	0.31
Myocardial infarction	7 (1.3)	7 (2.1)	0 (0.0)	** < 0.01**
TLR	11 (2.0)	11 (3.3)	0 (0.0)	** < 0.01**
Stroke	9 (1.6)	6 (1.8)	3 (1.4)	0.69
Cardiac decompensation	12 (2.2)	9 (2.7)	3 (1.4)	0.28

Data are presented as counts (%).* P*-values set in boldface indicate statistical significance. *CAD* coronary artery disease; *MACCE* major adverse cardiac and cerebrovascular events; *PCI* percutaneous coronary intervention; *TAVR* transcatheter aortic valve replacement; *TLR* target lesion revascularization

### Effect of PCI on outcomes after TAVR

#### Baseline characteristics

In 333 of 553 patients (60.2%) CAD was diagnosed according to the above-mentioned criteria. Of those, 120 patients (36.0%) received periprocedural PCI as part of the TAVR management (TAVR + PCI group) and 213 patients (64%) were treated with isolated TAVR (isolated TAVR group). Most baseline characteristics were comparable between the two groups and are presented in Table 3. Men constituted 55.0% in the TAVR + PCI group compared to 44.1% in the isolated TAVR group (*P* = 0.06). Cardiovascular risk factors and history of MI were similar distributed in both groups, resulting in an average EuroSCORE of 5.5 ± 4.0 vs. 6.6 ± 5.9 (*P* = 0.22) and a STS score of 6.1 ± 3.9 vs. 7.3 ± 5.0 (*P* = 0.13). Baseline SS was significantly higher in the TAVR + PCI group compared to the isolated TAVR group (14.9 ± 9.1 vs. 8.5 ± 9.1 (*P* < 0.001)). After PCI, SS decreased to 6.3 ± 6.7 in this group (residual SS). Among patients undergoing TAVR and PCI, the mean aortic valve area was significantly larger (0.7 ± 0.2 cm^2^ vs. 0.6 ± 0.2 cm^2^; *P* = 0.02) and the mean transaortic valvular gradient lower (32.7 ± 12.4 mmHg vs. 36.3 ± 15.6 mmHg; *P* = 0.15) compared to those with isolated TAVR. Presence of COPD was significantly more often in the isolated TAVR group compared to the TAVR + PCI group (60.6% vs. 42.9%; *P* = 0.002).

**Table 3:** Baseline characteristics of patients with AS and CAD as well as of patients with and without periprocedural PCI separately

	Patients with CAD (n = 333)	TAVR + PCI (n = 120)	Isolated TAVR (n = 213)	*P* value
Age (years)	81.4 ± 6.1	81.3 ± 6.0	81.4 ± 6.1	0.88
Male (%)	160 (48.0)	66 (55.0)	94 (44.1)	0.06
Body mass index (BMI; kg/m^2^)	26.6 ± 4.9	26.5 ± 4.7	26.7 ± 5.0	0.61
NYHA functional class	3.0 ± 0.8	3.0 ± 0.7	3.0 ± 0.8	0.33
CCS class	1.1 ± 1.4	0.9 ± 1.3	1.2 ± 1.5	0.14
Diabetes mellitus (%)	98 (29.4)	36 (30.0)	62 (29.1)	0.86
Prior myocardial infarction (%)	73 (21.9)	27 (22.5)	46 (21.6)	0.85
Previous TIA/stroke (%)	42 (12.6)	16. (13.3)	26 (12.2)	0.77
Carotid stenosis > 70% (%)	17 (5.1)	8 (6.7)	9 (4.2)	0.34
Atrial fibrillation (%)	138 (41.4)	49 (40.8)	89 (41.8)	0.87
COPD (%)	180 (54.1)	51 (42.9)	129 (60.6)	** < 0.01**
Severe PAH (%)	67 (20.1)	23 (19.2)	44 (20.8)	0.73
mPAP (mmHg)	30.0 ± 11.4	28.7 ± 12.7	30.8 ± 10.5	0.08
LVEF (%)	61.7 ± 22.1	63.5 ± 23.8	60.6 ± 21.0	0.26
Cardiac output (l/min)	4.8 ± 8.3	4.3 ± 1.3	4.0 ± 1.2	0.15
Severe heart failure (%)	21 (6.3)	10 (8.4)	11 (5.1)	0.25
PCWP (mmHg)	22.0 ± 9.9	20.5 ± 8.7	23.0 ± 10.6	0.22
Troponin T (ng/l)	93.3 ± 646	51.7 ± 104	115.7 ± 798	0.23
Renal insufficiency (%)	140 (42.0)	52 (43.3)	88 (41.3)	0.72
Creatinine level (mg/dl)	1.2 ± 0.7	1.2 ± 0.7	1.2 ± 0.8	0.94
Creatinine clearance (ml/min)	51.8 ± 19.7	53.1 ± 21.6	51.1 ± 18.6	0.69
Aortic valve area (cm^2^)	0.6 ± 0.22	0.7 ± 0.2	0.6 ± 0.2	**0.02**
MTPG (mmHg)	35.1 ± 14.7	32.7 ± 12.4	36.3 ± 15.6	0.15
EuroSCORE II (%)	6.2 ± 5.3	5.5 ± 4.0	6.6 ± 5.9	0.22
STS score	6.9 ± 4.7	6.1 ± 3.9	7.3 ± 5.0	0.13
SYNTAX score baseline	10.8 ± 8.8	14.9 ± 9.1	8.5 ± 7.9	** < 0.001**

Data are mean ± standard deviation or counts (%). *P*-values set in boldface indicate statistical significance.

*AS* aortic stenosis; *CAD* coronary artery disease; *CCS* Canadian Cardiovascular Society; *COPD* chronic obstructive pulmonary disease; *EuroSCORE* European System for Cardiac Operative Risk Evaluation; *LVEF* left ventricular ejection fraction; *mPAP* mean pulmonary arterial pressure; *mTPG* mean transaortic pressure gradient; *NYHA* New York Heart Association; *PAH* pulmonary arterial hypertension; *PCWP* pulmonary capillary wedge pressure; *STS* Society of Thoracic Surgeons; *TAVR* transcatheter aortic valve replacement; *TIA* transient ischemic attack

#### Clinical outcomes

**Fig. 2:**
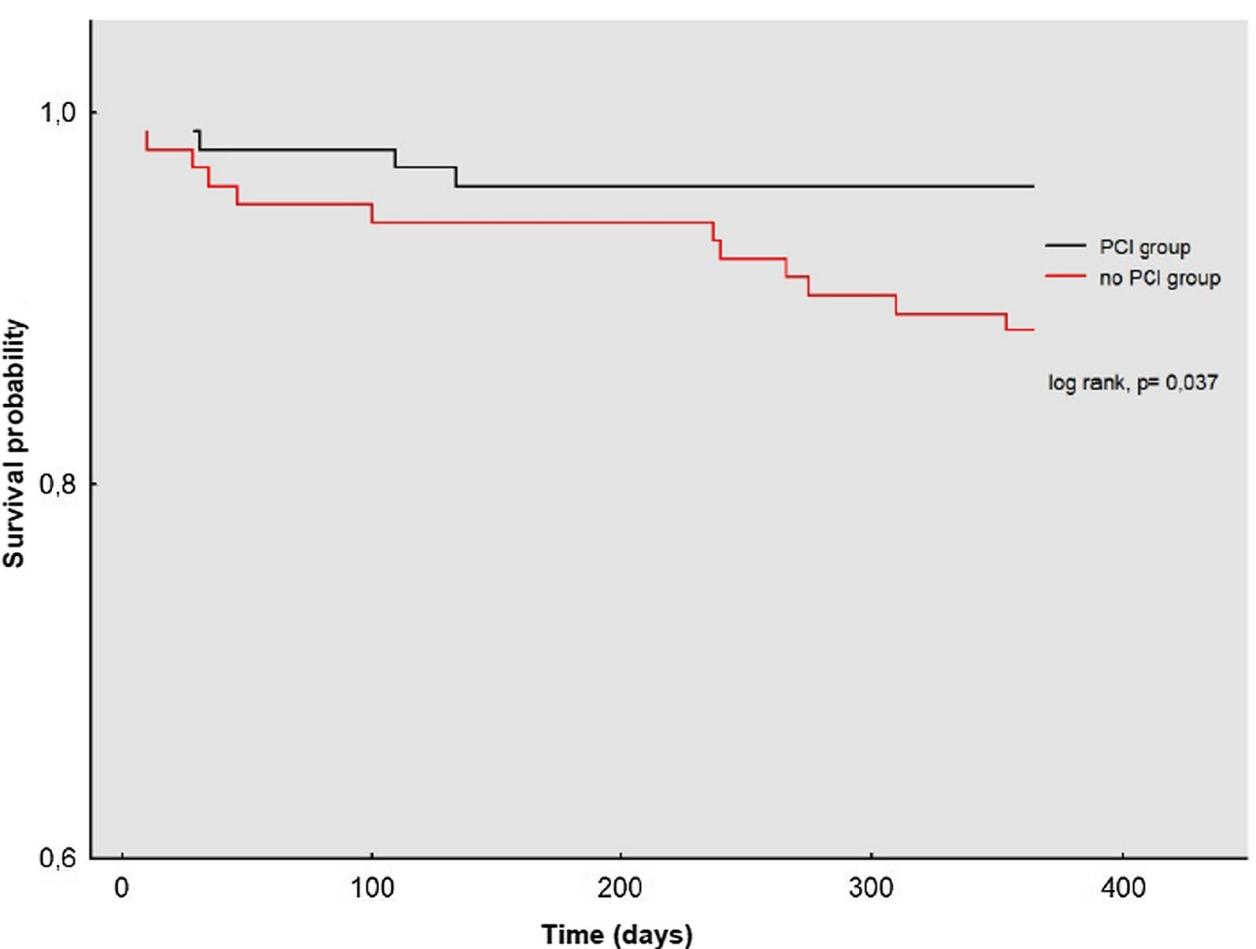
Kaplan–Meier curves for one-year mortality in the TAVR + PCI group compared to the isolated TAVR group. *PCI* percutaneous coronary intervention; *TAVR* transaortic valve replacement

One-year all-cause mortality was 7.5% among patients undergoing TAVR and PCI and was significantly lower compared to those treated with isolated TAVR (15.0%; *P* = 0.04). With regard to the 30-day mortality there was no significant difference between the two groups (1.7% vs. 4.2%; *P* = 0.19). Time-to-event curves obtained by KM for 1-year mortality are shown in Fig. 2. Moreover, there was a trend towards lower MACCE rates in the TAVR + PCI group, however without reaching statistically significance (18.3% vs. 24.4%, *P* = 0.20). In contrast, TLR rates were significantly increased in patients with additional PCI compared to patients with isolated TAVR (6.7% vs. 1.4%; *P* = 0.01). To adjust for differences between the TAVR + PCI group and the isolated TAVR group, a propensity score matching including all significant parameters was performed and validated our previous results. Baseline characteristics for the matched population are displayed in Additional file 1: Table 1. The clinical outcomes for the matched population are depicted in Table 4 and for the total cohort in Additional file 1: Table 2.

**Table 4:** One-year clinical outcomes after TAVR of propensity matched patients with CAD as well as of patients with and without periprocedural PCI separately

	Patients with CAD (n = 204)	TAVR + PCI (n = 102)	Isolated TAVR (n = 102)	*P* value
SYNTAX score residual	7.1 ± 7.0	6.0 ± 6.5	9.1 ± 7.9	**0.02**
One-year mortality	19 (9.3)	5 (4.9)	14 (13.7)	**0.03**
30-day mortality	5 (2.5)	2 (2.0)	3 (2.9)	0.65
MACCE	43 (21.1)	17 (16.7)	26 (25.5)	0.12
Myocardial infarction	6 (2.9)	3 (2.9)	3 (2.9)	1.00
TLR	9 (4.4)	7 (6.9)	2 (2.0)	0.08
Stroke	3 (1.5)	1 (0.9)	2 (2.0)	0.56
Cardiac decompensation	6 (2.9)	1 (0.9)	5 (4.9)	0.08

Data are presented as counts (%). *P*-values set in boldface indicate statistical significance. *CAD* coronary artery disease; *MACCE* major adverse cardiac and cerebrovascular events; *PCI* percutaneous coronary intervention; *TAVR* transcatheter aortic valve replacement; *TLR* target lesion revascularization

In a more detailed analysis, patients with high SS (> 22) were compared to patients with low SS (≤ 22). Hereby, we observed significantly higher rates of one-year mortality (29.4% vs. 10.4%) and MACCE (44.1% vs. 19.7%) in patients with SS > 22 (Additional file 1: Table 3). Similar results were seen when comparing patients with a residual SS < 8 and a value ≥ 8. Again, mortality and MACCE rates were significantly lower for patients with a residual SS < 8 (9.0% vs. 18.2% and 16.5% vs. 32.2%; Additional file 1: Table 4). In a further subgroup analysis, patients without CAD undergoing isolated TAVR were compared to patients with CAD undergoing TAVR + PCI and having a residual SS < 8 (Additional file 1: Table 5). Comparable rates were found for all examined clinical endpoints between the two groups.

**Table 5:** Results of multiple logistic regression analysis applied to assess associates of one-year mortality in the entire study population as well as in patients with AS and CAD undergoing TAVR

Characteristic	Chi-Square*	OR [95% CI)	*P* value
*Patients with AS and CAD*			
SYNTAX score residual ≥ 8	6.520	3.17 [1.31–7.70]	**0.011**
EuroSCORE ≥ 4%	2.651	2.41 [0.84–7.00]	0.103
Left bundle branch block after TAVR	6.743	3.39 [1.35–8.51]	**0.009**
Periprocedural PCI	3.507	0.37 [0.13–1.05]	0.061
*Entire study population*			
Severe pulmonary arterial hypertension	3.254	1.85 [0.95–3.43]	0.071
Left bundle branch block after TAVR	6.764	2.18 [1.21–3.92]	**0.009**
EuroSCORE ≥ 4%	5.653	2.18 [1.15–4.17]	**0.017**

*AS* aortic stenosis; *CAD* coronary artery disease; *CI* confidence interval; *EuroSCORE* European System for Cardiac Operative Risk Evaluation; *OR* odds ratio; *PCI* percutaneous coronary intervention; *TAVR* transcatheter aortic valve replacement

^*^The Wald chi-square values show the strength of association of variables with the risk for one-year mortality. *P*-values set in boldface indicate statistical significance

### Independent predictors of one-year mortality after TAVR for patients with CAD

In a first step, the subgroup of TAVR patients with concomitant CAD was analyzed. In this patient cohort, multivariate logistic regression analysis revealed residual SS ≥ 8 (OR = 3.17; *P* = 0.001)) and new onset of LBBB (OR = 3.39; *P* = 0.009) as independent predictors of one-year mortality following TAVR (Table 5). Periprocedural PCI was strongly associated with a reduced one-year mortality, however, without reaching statistical significance (*P* = 0.061).

In contrast, EuroSCORE ≥ 4% (OR = 2.18; *P* = 0.017) and new onset of LBBB (OR = 2.18; *P* = 0.009) represented independent predictors of an increased one-year mortality in the multivariate logistic regression analysis of the entire study population with 553 TAVR patients (Table 5).

## Discussion

The main findings of the present study may be summarized as follows: CAD is presented in two thirds of patients with severe AS undergoing TAVR. In total, patients with concomitant CAD suffered from significantly elevated MI rates within the first year after TAVR. Among patients with CAD, PCI as part of TAVR management was associated with an improved long-term outcome up to one-year compared to the conservative treatment group. In particular, a coronary revascularization strategy with a target SS < 8 was associated with significant lower rates of one-year mortality and MACCE. Thus, the residual SS displayed a strong predictor for an increased mortality risk after TAVR procedure in patients with concomitant CAD.

Concomitant coronary artery disease is highly prevalent among patients with severe aortic stenosis undergoing TAVR due to common pathophysiological processes with similar risk factors [1–3]. The reported prevalence ranges from 40 to 75%, depending on the criteria adopted for CAD diagnosis and with higher incidences in patients belonging to older age groups [18–21]. A mean SS of ~ 14 was recently reported in a series including 4.000 TAVR recipients with CAD [22]. These data are in accordance with our findings with almost two third of patients suffering from concomitant CAD and a mean baseline SS of ~ 11. The higher frequency of CAD in patients undergoing TAVR compared to SAVR (range 30–50%) can be explained by older age and more advanced atherosclerotic cardiovascular disease characterizing a generally high-risk population [23].

While CAD was demonstrated to negatively affect prognosis in patients undergoing SAVR [7–9], the impact of CAD on outcomes after TAVR is still a matter of debate [14]. Contradictory results have been reported in many former trials evaluating the association of CAD and clinical outcomes post TAVR [23–26]. Recently, a large meta-analysis by D´Ascenzo al. showed that the presence of CAD alone did not affect all-cause death in patients undergoing TAVR for severe AS [22]. In contrast, Sankaramangalam et al. observed in a meta-analysis of 15 studies that coexisting CAD did not impact on 30-day outcomes, but significantly increased all-cause mortality at one year [12]. Apart from a more frequent occurrence of myocardial infarction in the CAD cohort, the present trial demonstrated comparable one-year clinical outcomes after TAVR between patients with and without CAD in the entire study population. However, in a more detailed analysis, we observed that patients with a SS > 22 were linked to significantly higher rates of mortality and MACCE. These results highlight that CAD should not be addressed as a single pathological entity. The heterogenous nature of CAD, ranging from simple single-vessel to complex multivessel disease, results in a dilution of its prognostic effect, when pooling patients across the full spectrum of disease severity [15]. For this reason, more and more studies stratified TAVR patients according to CAD severity, mainly using the SS [15]. The SS is a well validated anatomical risk score which allows to quantify the severity of CAD and has been shown to risk-stratify patients with CAD as well as to predict long-term clinical outcomes in various subsets of patients undergoing PCI [27–29]. The large meta-analysis by D´Ascenzo et al. showed that higher values of SS were associated with worse short- and long-term outcomes in patients undergoing TAVR as well [22]. As also shown in our study, especially patients with a SS > 22 suffered from an increased one year-mortality and adverse events.

Furthermore, it was shown that more complete revascularization pre-TAVR assessed by the residual SS mitigated the risk exerted by CAD in patients undergoing TAVR [15]. Paradoxically, in former trials especially patients with higher SS often received less complete revascularization [30]. The present study demonstrates that a residual SS ≥ 8 is associated with a significantly increased risk of mortality and MACCE up to one year after TAVR. Moreover, our results suggest that the residual SYNTAX score represents a strong predictor for an increased mortality risk after TAVR procedure in patients with concomitant CAD. A residual SS of ≥ 8 was related to a threefold increased mortality risk. Our results are underlined by several large meta-analyses revealing the residual SS as the most appropriate measure for stratifying patients with CAD. Incomplete coronary revascularization and/or high residual SS were shown to negatively impact prognosis after TAVR. In this regard, Witberg et al. demonstrated in 3.107 patients undergoing TAVR that incomplete revascularization with residual SS > 8 was associated with an increased risk for mortality when compared to patients with no CAD or those with revascularization and residual SS < 8 [15]. Likewise, D´Ascenzo et al. could show in over 8.000 TAVR patients that PCI with a residual SS less than 8 reduced the one-year risk of death after TAVR [22].

Despite these findings, numerous former studies failed to demonstrate a clear benefit of PCI in the TAVR management and revealed comparable clinical outcomes in patients undergoing TAVR $+$ PCI versus isolated TAVR [31–33]. These results have generally been interpreted as a proof of feasibility and safety of PCI during the TAVR setting [34]. Current guidelines state that PCI should be considered in patients with a primary indication to undergo TAVR and with coronary artery diameter stenosis > 70% in proximal segments (class IIa level C recommendation) [13]. Our analysis demonstrates a benefit of periprocedural PCI with regard to long-term outcomes up to one year after TAVR, even after adjustment for confounders. Patients with successful PCI as part of TAVR management were associated with a reduced risk of one-year mortality and showed a trend towards lower MACCE rates compared to those with isolated TAVR. It should be emphasized that SS at baseline was significantly higher in patients undergoing TAVR and PCI compared to those with isolated TAVR (14.1 vs. 9.1; *P* < 0.001), whereas the residual SS was significantly lower (6.0 vs. 9.1; *P* = 0.02). Moreover, after successful PCI patients with CAD and a residual SS < 8 were non-inferior for one-year prognosis compared to patients without CAD, although they often possess higher risk profiles at baseline [29].

Nevertheless, in our retrospective study we cannot rule out that the results were partly influenced by the fact that other criteria besides the SS score were also included in the decision-making process to carry out PCI. In this context it is certainly possible that some patients with CAD did not receive PCI due to poorer health conditions being associated with a poorer one-year survival as they presumably would not have benefited from it in the long term. Even if we have tried to counteract this bias as best as possible through propensity score matching analysis and multivariate logistic regression analysis, randomized studies like the ongoing *Percutaneous Coronary Intervention Prior to Transcatheter Aortic Valve Implantation* (ACTIVATION) trial (ISRCTN75836930) or the *Nordic aortic valve intervention (NOTION) -3* trial (NCT03058627) are urgently necessary to prove our findings [35]. Nevertheless, our results clearly indicate that a residual SS-guided revascularization strategy improves prognosis after TAVR. Patients with more severe CAD could be those who benefit the most from coronary revascularization as part of TAVR management. Considering that approximately 50% of TAVR patients suffer from concomitant CAD and the fact that TAVR population is getting increasingly younger, the residual SS could represent a useful tool to select these patients who really benefit from coronary revascularization and to improve their prognosis.

Interestingly, we further demonstrated that the group with a successful PCI showed similar rates of myocardial infarction post TAVR when compared to the group without PCI representing with an initial significantly lower SS. Moreover, patients with CAD undergoing PCI and having a residual SS < 8 showed even similar rates of myocardial infarction post TAVR compared to patients without CAD. Coronary events after TAVR are common. A former study revealed that approximately one-tenth of patients undergoing TAVR were readmitted for an acute coronary syndrome (ACS) after a median follow up of ~ 2 years [36]. The potential mechanisms are multifarious, but a majority of coronary events are likely related to an atherothrombotic mechanism by a progression of CAD [33]. Prior CAD was already shown to be a risk factor for ACS following TAVR [36]. Our results imply that successful PCI as part of TAVR management prevents coronary events after TAVR, but of course further randomized studies are necessary to confirm the thesis. Taking into account that the occurrence of ACS is associated with impaired prognosis and higher mortality after TAVR, physicians should consider PCI properly, when evaluating and assessing TAVR candidates with coexisting CAD [36].

### Limitations

The results of our study have to be interpreted with several confinements: Our analysis is a single-center retrospective observational study with all the bias ascribed to such type of design. Treatment strategy including indication for coronary revascularization and extent of revascularization was performed according to the Heart Team decision and was not randomized. However, SS was calculated for each patient and has proven to be good marker of need for revascularization in former trials. Furthermore, we performed a propensity score matching to eliminate possible confounders. Nevertheless, other criteria such as multimorbidity or severe illness may also have contributed to the decision-making process and it cannot be ruled out that some patients with CAD did not receive PCI for example due to poorer health conditions being associated with poorer one-year survival as they presumably would not have benefited from it in the long term. Next, coronary revascularization was performed within a period of 6 months prior and 3 months following TAVR. Therefore, we cannot make a statement about the optimal timing for coronary revascularization as part of TAVR management. Lastly, our clinical follow-up was restricted to 12 months.

## Conclusion

Our results indicate that a residual SS-targeted revascularization strategy improves prognosis after TAVR in patients with concomitant CAD. The SS represents a useful tool to select those patients who could benefit from a periprocedural coronary revascularization. PCI aiming for a residual SS < 8 was associated with improved one-year clinical outcomes. Considering the worldwide trend of treating lower-risk patients with TAVR, our findings can contribute to achieve optimal outcomes in this important subgroup, which totals almost two-thirds of patients undergoing TAVR. Adequately powered randomized trials investigating long-term clinically relevant outcomes are required to determine the best management strategy for CAD in TAVR population.

## Supplementary Information

**Additional file 1**. Supplementary data.

## Data Availability

The datasets used and/or analysed during the current study are available from the corresponding author on reasonable request.

## Abbreviations

ACS: Acute coronary syndrome
AS: Aortic stenosis
AVA: Aortic valve area
CABG: Coronary artery bypass graft
CAD: Coronary artery disease
CCS: Canadian Cardiovascular Society
COPD: Chronic obstructive pulmonary disease
EuroSCORE: European System for Cardiac Operative Risk Evaluation
KM: Kaplan Meier
LBBB: Left bundle branch block
LVEF: Left ventricular ejection fraction
MACCE: Major cardiovascular and cerebrovascular events
MI: Myocardial infarction
mPAP: Mean pulmonary arterial pressure
mTPG: Mean transaortic pressure gradient
NYHA: New York Heart Association
OR: Odds ratio
PAH: Pulmonary arterial hypertension
PCI: Percutaneous coronary intervention
PCWP: Pulmonary capillary wedge pressure
SAVR: Surgical aortic valve replacement
SD: Standard deviation
SS: SYNTAX score
STS: Society of Thoracic Surgeons
SYNTAX: Synergy Between PCI With Taxus and Cardiac Surgery
TAVR: Transcatheter aortic valve replacement
TIA: Transient ischemic attack
TLR: Target lesion revascularization
