# Supplementary material for: Impact of extent of coronary artery disease and percutaneous revascularization assessed by the SYNTAX score on outcomes following transcatheter aortic valve replacement
Source: BMC Cardiovasc Disord. 2021 Nov 30;21:568. doi: 10.1186/s12872-021-02374-y (PMC8638523; doi:10.1186/s12872-021-02374-y)
Supplement: Supplementary file 1 — Additional file 1. Supplementary data. [file 12872_2021_2374_MOESM1_ESM.docx]

**Supplementary data**

| **Supplementary table 1** Baseline characteristics of propensity matched patients with AS and CAD | | | | | | | |
| --- | --- | --- | --- | --- | --- | --- | --- |
|  | Patients with CAD  (n=204) | | TAVR+PCI  (n=102) | | Isolated TAVR  (n=102) | | P value |
| Age (years) | | 81.7±6.3 | | 81.3±6.1 | | 82.1±6.5 | 0.37 |
| Male (%) | | 104 (51.0) | | 52 (51.0) | | 52 (51.0) | 1.00 |
| Body mass index (BMI; kg/m²) | | 26.4±4.7 | | 26.5±4.7 | | 26.3±4.7 | 0.79 |
| NYHA functional class | | 3.0±0.8 | | 2.9±0.7 | | 3.0±0.8 | 0.22 |
| CCS class | | 1.0±1.4 | | 1.0±1.3 | | 1.0±1.4 | 0.88 |
| Diabetes mellitus (%) | | 67 (32.8) | | 34 (33.3) | | 33 (32.4) | 0.88 |
| Prior myocardial infarction (%) | | 38 (18.6) | | 21 (20.6) | | 17 (16.7) | 0.47 |
| Previous TIA/stroke (%) | | 24 (11.8) | | 12 (11.8) | | 12 (11.8) | 1.00 |
| Carotid stenosis > 70% (%) | | 11 (5.4) | | 7 (6.9) | | 4 (3.9) | 0.35 |
| Atrial fibrillation (%) | | 75 (36.8) | | 40 (39.2) | | 35 (34.3) | 0.47 |
| COPD (%) | | 90 (44.1) | | 43 (42.2) | | 47 (46.1) | 0.57 |
| Severe PAH (mmHg) | | 38 (18.6) | | 18 (17.7) | | 20 (19.6) | 0.72 |
| mPAP (mmHg) | | 28.8±11.5 | | 28.0±12.6 | | 30.0±10.0 | 0.16 |
| LVEF (%) | | 61.3±21.5 | | 63.3±23.8 | | 59.2±18.7 | 0.35 |
| Cardiac output (l/min) | | 5.4±11.0 | | 4.3±1.4 | | 4.2±1.3 | 0.61 |
| Severe heart failure (%) | | 15 (7.3) | | 9 (8.8) | | 6 (5.9) | 0.42 |
| PCWP (mmHg) | | 20.6±9.4 | | 19.9±9.0 | | 21.6±10.0 | 0.63 |
| Troponin T (ng/l) | | 114.6±793 | | 51.5±109 | | 167.6±1070 | 0.26 |
| Renal insufficiency (%) | | 83 (40.7) | | 43 (42.2) | | 40 (39.2) | 0.67 |
| Creatinine level (mg/dl) | | 1.3±0.8 | | 1.2±0.7 | | 1.3±0.9 | 0.82 |
| Creatinine clearance (ml/min) | | 51.8±20.5 | | 53.1±21.3 | | 50.6±19.8 | 0.53 |
| Aortic valve area (cm^2^) | | 0.7±0.2 | | 0.7±0.2 | | 0.7±0.2 | 0.63 |
| MTPG (mmHg) | | 33.6±13.4 | | 32.8±12.7 | | 34.3±14.1 | 0.61 |
| EuroSCORE II (%) | | 5.9±5.3 | | 5.5±3.9 | | 6.5±6.5 | 0.75 |
| STS score | | 6.7±4.6 | | 6.0±3.8 | | 7.3±5.1 | 0.20 |
| SYNTAX score baseline | | 11.6±8.4 | | 14.1±8.2 | | 9.1±7.9 | **<0.001** |
| SYNTAX score residual | | 7.1±7.0 | | 6.0±6.5 | | 9.1±7.9 | **0.02** |

Data are mean ± standard deviation or counts (%).

AS, aortic stenosis; CAD, coronary artery disease; CCS, Canadian Cardiovascular Society; COPD, chronic obstructive pulmonary disease; EuroSCORE, European System for Cardiac Operative Risk Evaluation; LVEF, left ventricular ejection fraction; mPAP; mean pulmonary arterial pressure; mTPG, mean transaortic pressure gradient; NYHA, New York Heart Association; PAH, pulmonary arterial hypertension; PCWP, pulmonary capillary wedge pressure; STS, Society of Thoracic Surgeons; TAVR, transcatheter aortic valve replacement; TIA, transient ischemic attack.

| **Supplementary table 2** One-year clinical outcomes after TAVR of patients with CAD as well as of patients with and without periprocedural PCI separately (unmatched study population). | | | | |
| --- | --- | --- | --- | --- |
|  | Patients with CAD  (n=333) | TAVR+PCI  (n=120) | Isolated TAVR  (n=213) | P value |
| SYNTAX score residual | 7.2±7.3 | 6.3±6.7 | 8.5±7.9 | 0.06 |
| One-year mortality | 41 (12.3) | 9 (7.5) | 32 (15.0) | **0.04** |
| 30-day mortality | 11 (3.3) | 2 (1.7) | 9 (4.2) | 0.19 |
| MACCE | 74 (22.2) | 22 (18.3) | 52 (24.4) | 0.20 |
| Myocardial infarction | 7 (2.1) | 3 (2.5) | 4 (2.2) | 0.71 |
| TLR | 11 (3.3) | 8 (6.7) | 3 (1.4) | **0.01** |
| Stroke | 6 (1.8) | 1 (0.8) | 5 (2.4) | 0.29 |
| Cardiac decompensation | 9 (2.7) | 1 (0.8) | 8 (3.8) | 0.08 |

Data are presented as counts (%). CAD, coronary artery disease; MACCE, major adverse cardiac and cerebrovascular events; PCI, percutaneous coronary intervention; TAVR, transcatheter aortic valve replacement; TLR, target lesion revascularization.

| **Supplementary table 3** Comparison of one-year clinical outcomes after TAVR of patients with CAD and low SS (≤22) vs high SS (>22) at baseline | | | |
| --- | --- | --- | --- |
|  | Low SS (baseline)  (n=299) | High SS (baseline)  (n=34) |  |
| Death | 31 (10.4) | 10 (29.4) |  |
| MACCE | 59 (19.7) | 15 (44.1) |  |
| Myocardial infarction | 5 (1.7) | 2 (5.9) |  |
| TLR | 10 (3.3) | 1 (2.9) |  |
| Stroke | 6 (2.0) | 0 (0.0) |  |
| Cardiac decompensation | 7 (2.3) | 2 (5.9) |  |

Data are presented as counts (%). CAD, coronary artery disease; MACCE, major adverse cardiac and cerebrovascular events; PCI, percutaneous coronary intervention; TAVR, transcatheter aortic valve replacement; SS, SYNTAX score; TLR, target lesion revascularization.

| **Supplementary table 4** Comparison of one-year clinical outcomes after TAVR of patients with CAD and low residual SS (< 8) vs. high residual SS (≥ 8) | | | |
| --- | --- | --- | --- |
|  | SS < 8 (residual)  (n=212) | SS ≥ 8 (residual)  (n=121) |  |
| MACCE | 35 (16.5) | 39 (32.2) |  |
| Death | 19 (9.0) | 22 (18.2) |  |
| Myocardial infarction | 3 (1.4) | 4 (3.3) |  |
| TLR | 4 (1.9) | 7 (5.8) |  |
| Stroke | 4 (1.9) | 2 (1.7) |  |
| Cardiac decompensation | 5 (2.4) | 4 (3.3) |  |

Data are presented as counts (%). CAD, coronary artery disease; MACCE, major adverse cardiac and cerebrovascular events; SS, SYNTAX score; TAVR, transcatheter aortic valve replacement; TLR, target lesion revascularization.

| **Supplementary table 5** One-year clinical outcomes of patients without CAD undergoing isolated TAVR compared to patients with CAD undergoing TAVR+PCI and having a residual SS < 8. | | | | |
| --- | --- | --- | --- | --- |
|  | Overall population  (n=301) | PCI +SS < 8 (residual)  (n=81) | No CAD  (n=220) |  |
| One-year mortality | 38 (12.6) | 3 (3.7) | 35 (15.9) |  |
| 30-day mortality | 5 (1.7) | 0 (0.0) | 5 (2.3) |  |
| MACCE | 49 (16.2) | 8 (9.9) | 41 (18.6) |  |
| Myocardial infarction | 1 (0.3) | 1 (1.2) | 0 (0.0) |  |
| TLR | 3 (1.0) | 3 (3.7) | 0 (0.0) |  |
| Stroke | 3 (1.0) | 0 (0.0) | 3 (1.4) |  |
| Cardiac decompensation | 4 (1.3) | 1 (1.2) | 3 (1.4) |  |

Data are presented as counts (%). CAD, coronary artery disease; MACCE, major adverse cardiac and cerebrovascular events; PCI, percutaneous coronary intervention; SS, SYNTAX score; TAVR, transcatheter aortic valve replacement; TLR, target lesion revascularization.
